# K-SPMM: a database of murine spermatogenic promoters modules & motifs

**DOI:** 10.1186/1471-2105-7-238

**Published:** 2006-05-03

**Authors:** Yi Lu, Adrian E Platts, G Charles Ostermeier, Stephen A Krawetz

**Affiliations:** 1 Department of Computer Science, Wayne State University, 5143 Cass Avenue, 431 State Hall, Detroit, MI 48202, USA; 2 Applied Genomics Technologies Center, Bioinformatics Group, BioSciences, 5047 Gullen Mall, Detroit, MI 48202, USA; 3 Department of Obstetrics and Gynecology, Wayne State University, 275 E. Hancock, Detroit, MI, 48201, USA; 4 Center for Molecular Medicine and Genetics, Wayne State University School of Medicine, 5240 Eugene Applebaum Building, 259 Mack Avenue, Detroit, MI 48201, USA; 5 Institute for Scientific Computing, Wayne State University, 275 E. Hancock, Detroit, MI, 48201, USA

## Abstract

**Background:**

Understanding the regulatory processes that coordinate the cascade of gene expression leading to male gamete development has proven challenging. Research has been hindered in part by an incomplete picture of the regulatory elements that are both characteristic of and distinctive to the broad population of spermatogenically expressed genes.

**Description:**

*K-SPMM*, a database of murine **S**permatogenic **P**romoters **M**odules and **M**otifs, has been developed as a web-based resource for the comparative analysis of promoter regions and their constituent elements in developing male germ cells. The system contains data on 7,551 genes and 11,715 putative promoter regions in Sertoli cells, spermatogonia, spermatocytes and spermatids. *K-SPMM *provides a detailed portrait of promoter site components, ranging from broad distributions of transcription factor binding sites to graphical illustrations of dimeric modules with respect to individual transcription start sites. Binding sites are identified through their similarities to position weight matrices catalogued in either the JASPAR or the TRANSFAC transcription factor archives. A flexible search function allows sub-populations of promoters to be identified on the basis of their presence in any of the four cell-types, their association with a list of genes or their component transcription-factor families.

**Conclusion:**

This system can now be used independently or in conjunction with other databases of gene expression as a powerful aid to research networks of co-regulation. We illustrate this with respect to the spermiogenically active protamine locus in which binding sites are predicted that align well with biologically foot-printed protein binding domains.

**Availability:**

## Background

K-Means and hierarchical clustering analyses are increasingly used in microarray studies to reveal correlated expression between groups of genes. Through time-series experiments, regimes of co-expression and regulatory cascades have been described. Nonetheless, determining the mechanistic relationship underlying co-regulation has not been trivial. The subtle interplay of systems controlling expression makes hidden variable models attractive to the analyst but ultimately problematic for the biologist seeking verifiable pathways. Studies of co-regulation have been most effective in bridging this gap when gene expression data has been used in conjunction with data describing transcription factor specificity. The approach allows the agents and outcomes of regulation to be explicitly connected [1].

Transcriptional mechanisms regulating expression are currently thought to include binary differentiating systems that potentiate chromatin for transcription as well as a scalar mechanism that determines the extent and products of expression. The binding of transcription factors within critically defined promoter regions is thought to be a class of scalar regulation that initiates transcription only after binary control mechanisms have potentiated the chromatin locus [2]. Reverse engineering systems of co-regulation using arrays alone has been complicated by the morphological complexity of the sites to which transcription factors bind in these regions. These occur as higher order compound modules where an assortment of agonistic interactions can occur between transcription factors binding at different constituted locations. The compilation of detailed maps covering the functionally active elements in any given cell type can be aided by mapping sequence conservation between species. In this research the progression of germ cells of mouse and four other vertebrates (rat, dog, chicken and human) through spermatogenesis show a sufficiently similar development to make conservation a useful indicator of sequence significance.

The differentiation of cells in the testes occurs continuously in adult mice through the serial interplay of gene expression that affects approximately one third of the genome. This includes an estimated 4% of genes that are uniquely expressed during spermatogenesis [3]. Spermatogenesis begins amongst spermatogonia immersed within a population of Sertoli 'nurse' cells. Spermatogonia mature through spermatocytogenesis into spermatocytes towards an extended meiotic division. Subsequently, post-meiotic round spermatids are formed that differentiate to attain species-specific elongated spermatozoa. This well characterised serial differentiation [4-6] makes the cells well-suited for the study of gene expression with respect to promoter structure. The software outlined in this communication, K-SPMM (**K**rawetz-Lab database of **S**permatogenic **P**romoters **M**odules &**M**otifs), provides online access to a suite of promoter structure-based analytical tools. This employs a database of known transcriptional control elements as an *in-silico *discovery tool that is targeted to the promoter regions of a set of testes expressed genes that regulate male germ cell differentiation.

### Construction & content

A dataset of spermatogenically active genes was gathered from nine NCBI published cDNA libraries [7] and accessed on or before December 25^th^, 2005. The libraries, representing four major cell-types found within the testes, were selected as follows: Sertoli (lib#-12732, 11283), spermatogonia (lib#-6789, 6788, 11285), spermatocytes (lib#-6787, 11284, 11128) and spermatids (lib#-6786). Their respective promoter sequences were downloaded from mm5 genome build of DBTSS, the DataBase of Transcription Start Sites [8] and were used to generate the three *K-SPMM *databases. These databases describe murine promoter location, Transcription Factor Binding Site (TFBS) distribution and the location of putative homo or heterodymeric transcription-factor modules. This data was enhanced with a per-base conservation score relative to four vertebrate genomes hg17, rn3, canFam1 and galGal2 obtained from the UCSC archive of phastCons scores [9] that were averaged on a per-module basis.

The promoter location database contains the many-to-one mapping of 11,715 potential promoter regions with the 7,551 genes in the cDNA libraries. Each DBTSS promoter sequence contains a 1 kb upstream sequence from each Transcription Start Site (TSS) described. Analysis of the 200 bp sequence downstream from TSS is available as an optional element. Annotation of the genes associated with each promoter was extracted from NIH DAVID 2.1 [10]. The TFBS population within each promoter is generated based on the models described in JASPAR [11] and Transfac [12]. JASPAR models are the default selection. These are derived from 81 biologically verified PWMs, Position Weight Matrices matched with a lower threshold p value of 0.98 for a match. This yields 422,027 TFBSs for the 11,715 promoters, with an average of 36 binding sites per promoter. Transfac models are available as an alternative. They include 236 PWMs derived from mouse, rat and human matched to promoter regions with a lower threshold p value of 0.96. The lower threshold of detection (p value) is adjusted for each database to qualitatively reflect differences in PWM design. These include length and specificity of matrices. Matrix matching is reported in conjunction with TFBS family data in order to identify a specific TFBS family member. The Transfac option is currently available as a beta version, extending the range and specificity of the binding motifs contained within JASPAR. Nonetheless while the response elements of generic transcription factors are now well represented neither database is as yet fully complete with respect to the binding sequence of those transcription factors that have been shown to direct spermatogenesis.

Transcription factor binding sites were then refined and combined on the basis of distance metrics [13]. This identified 217,554 potential multi-TFBS module sites using the transcription factor combinations from JASPAR models and 593,094 module sites from the Transfac models. Each module is named to reflect the binary combination of its component transcription factor families. For example, ZBPF-ETSF identifies a module combining a zinc-binding protein factor site and a murine ETS1 factor binding site. Not all possible modules were discovered. Using the JASPAR matrices, only 1,588 of approximately 6,500 possible binary modules were mapped.

The system is executed as a JSP application within a Jakarta Tomcat framework with SQL queries directed to a local MySQL database.

### Utility

**Figure 1:**
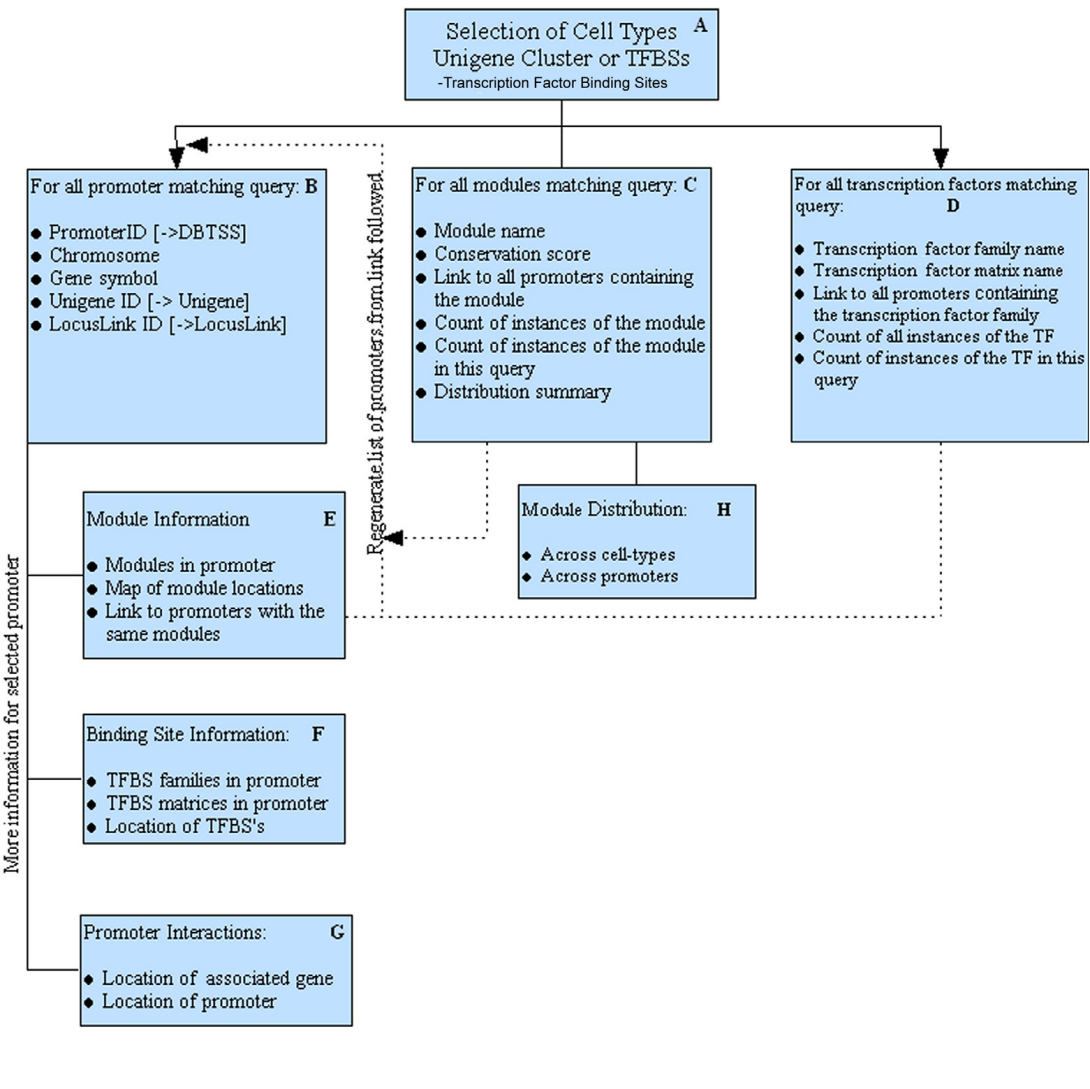
The system user-interface design. Panels [A-H] show the data available through a typical search strategy.

**Figure 2:**
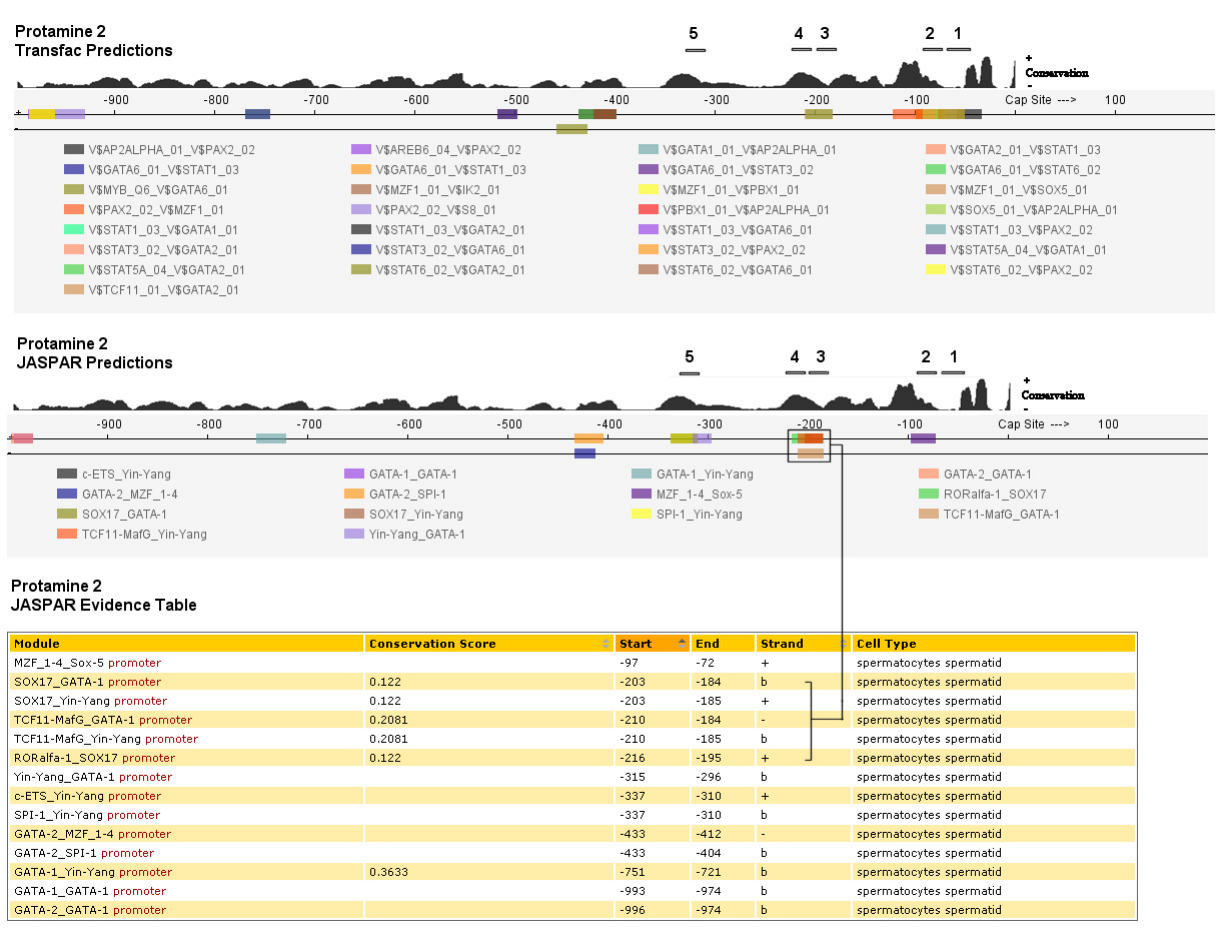
The module output for the promoter region of Protamine 2. The graphical output for Transfac PWMs is shown above the graphical and tabular output for JASPAR in which one potential binding domain has been highlighted. Regions noted as 1–5 have been added and correspond to the location of *in vitro *protected foot-printed sites proximate to the TSS [23]. An alternate representation of sequence conservation within the domain has been included from the vertebrate conservation track from the UCSC Genome Browser. This illustrates more fully the extent of alignment between conserved nucleotides and the predicted binding sequences, as such it is similar to the conservation data tabulated for four comparator species in the evidence table.

As shown in Fig. 1, K-SPMM is designed to assist the user in rapidly characterizing sub-populations of differentially partitioned promoter elements. Through an initial query (Fig. 1A) the user identifies promoter elements common to, excluded from or exclusive to any of the 4 cell-types. Alternatively the system can be queried to search for promoters associated with a defined list of genes or specific TFBS families. These points of initiation can be combined to identify promoters that exhibit similar TFBS components, gene association and expression in a given cell-type. Search results can be presented as a function of matching promoters (Fig. 1B), matching modules (Fig. 1C) or matching TFBSs (Fig. 1D), thus facilitating inquiry from any of the analytical perspectives. Additional information describing the locations of the modules relative to the TSS, as well as the distribution of the transcription factors as a function of cell type is provided (Fig. 1E–H). A promoter map shows the locations of modules alongside the level of module conservation (Fig. 2). Internal links are provided to further refine the promoter regions based upon shared components while external links are provided to NCBI and DBTS databases to contextualise the genomic locations discovered. Data from any of the system's components can be viewed online or downloaded as Excel, XML or delimited files.

## Discussion

The response of the genome to spermatogenic differentiation is global, affecting the expression of approximately one third of its genes. Many of these genes are expressed as tissue specific isoforms [3] or are derived from the use of alternative promoters [14]. Their expression is coordinated through the use of a suite of spermatogenic-variants of general transcriptional factors. Examples include, TFIIA-tau, the testis-specific transcription factor IIA [15], TAF, the TBP-associated factor [16], TRF2, the TBP-related factor 2 [17], TAF7L a paralog of transcription factor TFIID subunit [18] and ATF, the TFIIA alpha/beta-like factor [19]. Several non germ-cell specific factors like TBP, the TATA-binding protein, TFIIB and RNA polymerase II, accumulate to a greater extent in germ cells than they do in any other somatic cell type [20]. Together, these properties of the spermatogenic system provide a unique model to dissect the complex and unique regulatory transcription factor mechanistic network that governs the expression of male germ cells.

The protamine locus provides a key example of a gene cluster that is active in the latter spermiogenic phase of spermatogenesis. It contains both protamine genes (P*rm1 *& P*rm2*) required for the successful repackaging of nuclear DNA into the spermatozoon nucleus as well as one of the condensation enabling genes (T*np2*). The coordinate regulation of this locus has been widely investigated [21-31]. Upstream promoter regions of the genes have been annotated for their conservation and potential for transcription factor binding [32] including DNAse-1 footprinted regions indicative of protein binding sites [23].

Exploring each gene in turn with respect to predictions made by the K-SPMM system and restricting our analysis to those regions that have also been annotated reveals much of biological interest. P*rm2 *has three potential binding domains as determined by DNAse-1 footprinting in the annotated region. Transfac predictions were observed in all 3 binding domains and in two of the three regions using JASPAR models (Fig. 2). These included the SRY (5' AACAAT 3') binding site that has been previously reported [32] as well as YY1 & GATA1 (5' CCAT 3' & 5' ACAATGA 3') binding sites. It is noteworthy that several YY1-GATA1 modules were also identified in more distal protected and conserved regions.

One of the few sites identified in the upstream region of the T*np2 *gene was YY1. This reflects the lack of candidate factors that were identified in sufficiently close proximity to form an active dimeric. Nevertheless, where biological evidence suggests a region of interest, it is possible (Fig. 1-F) to manually examine all binding factors for candidate modules.

In the 200 bp upstream region of P*rm1 *three potential modules are reported using the JASPAR PWMs. The first S8-GATA module has moderate 20 to 40 percent conservation relative to four comparator organisms and overlaps a region highlighted in the annotation as having a potential for TFBS binding. More interestingly, at approximately 87 bp upstream of TSS lies a moderately conserved YY1 doublet (5' CCAT 3'/5' ATGG 3') overlapping on opposite strands and paired with a third YY1 site located 20 bp further upstream. This places all three YY1 elements within the 113 bp upstream region required for P*rm1 *expression [28], with the second YY1 element within the -110 to -150 region determined to be necessary for testis specific expression. The YY1 element, while ubiquitous, is one of the known factors to be found upstream of a considerable number of spermiogenic genes [33]. Transfac predictions supported the JASPAR predictions with some differences in nomenclature noted.

CREM, the cAMP Response Element Modulator that directly binds to CRE, has been widely implicated for its role in spermiogenesis. CREM deficient mice arrest spermatogenesis at the early round spermatid stage [34], with the gene structure and function of CREM notably conserved between mouse and man [35]. In somatic cells, activation by CREM requires phosphorylation of Ser_117 _and interaction with CBP, the ubiquitous CREB-Binding Protein co-activator. By contrast, the transcriptional activity of CREM in testes is controlled through its interaction with ACT, the tissue-specific Activator of CREM in Testis [36,37] that is regulated by the testis-enriched kinesin KIF17b [38]. Interestingly, CREMtau binding sites have been identified upstream of PRM1 and PRM2[32] and in conjunction with the GCNF response element [21] but these occur only as half-site CRE motifs with several mismatches to the core consensus sequence. The current implementation of the software employs a rigorous approach to PWM matching to minimize reporting false-positive candidates. Accordingly, although CREMtau sites are of interest, they are examples of candidate sites that in the current system fall below the binding confidence criteria.

## Conclusion

Together these results show that the use of transcription factor colocalization in conjunction with conservation as implemented in the K-SPMM promoter discovery tool yield potential sites of transcription factor binding that are biologically well validated. In testing the system, we noted the presence of the YY1 response element in the upstream regions of all three genes in the protamine domain that has been associated with a sterol response element binding protein that regulates proacrosin, another haploid expressed gene. [39]. The developmentally significant GATA family of elements were also over-represented in biologically significant locations in two of the three genes. This illustrates well how K-SPMM can be used to inform the process of biological validating functional binding elements.

## Abbreviations

DBTSS The **D**ata**b**ase of **T**ranscription **S**tart **S**ites [40]

JASPAR An open source database of transcription factor DNA-binding preferences [41]

K-SPMM **K**rawetz-Lab database of **S**permatogenic **P**romoters **M**odules &**M**otifs [42]

NIH DAVID National Institute of Health DAVID Gene Annotation system [43]

PWM **P**osition **W**eight **M**atrix

TFBS **T**ranscription **F**actor **B**inding **S**ite

Transfac The **Trans**cription **Fac**tor Database [44]

TSS **T**ranscription **S**tart **S**ite

UCSC Genome Browser University of California Genome Browser [45]

## Authors' contributions

YL developed the final version of the JSP codebase and constructed the SQL database. AEP coordinated the bioinformatic investigation leading to the data used in the system. GCO provided biological context and guidance during the initial phase of system development. SAK originated the concept and supervised its design and implementation. The manuscript was drafted by AEP and SAK with the input and approval of all authors.
